# Radiomic texture analysis based on neurite orientation dispersion and density imaging to differentiate glioblastoma from solitary brain metastasis

**DOI:** 10.1186/s12885-023-11718-0

**Published:** 2023-12-14

**Authors:** Jie Bai, Mengyang He, Eryuan Gao, Guang Yang, Hongxi Yang, Jie Dong, Xiaoyue Ma, Yufei Gao, Huiting Zhang, Xu Yan, Yong Zhang, Jingliang Cheng, Guohua Zhao

**Affiliations:** 1 Department of Magnetic Resonance Imaging, The First Affiliated Hospital of Zhengzhou University, No. Jianshe Dong Road, Zhengzhou, 450052 China; 2 School of Cyber Science and Engineering, Zhengzhou University, Zhengzhou, 450001 China; 3 Shanghai Key Laboratory of Magnetic Resonance, East China Normal University, Shanghai, 200062 China; 4 School of Information Engineering, North China University of Water Resources and Electric Power, Zhengzhou, 450046 China; 5 MR Research Collaboration, Siemens Healthineers, Wuhan, 201318 China

**Keywords:** Glioblastoma, Solitary brain metastasis, Radiomic texture analysis, NODDI

## Abstract

**Background:**

We created discriminative models of different regions of interest (ROIs) using radiomic texture features of neurite orientation dispersion and density imaging (NODDI) and evaluated the feasibility of each model in differentiating glioblastoma multiforme (GBM) from solitary brain metastasis (SBM).

**Methods:**

We conducted a retrospective study of 204 patients with GBM (n = 146) or SBM (n = 58). Radiomic texture features were extracted from five ROIs based on three metric maps (intracellular volume fraction, orientation dispersion index, and isotropic volume fraction of NODDI), including necrosis, solid tumors, peritumoral edema, tumor bulk volume (TBV), and abnormal bulk volume. Four feature selection methods and eight classifiers were used for the radiomic texture feature selection and model construction. Receiver operating characteristic (ROC) curve analysis was used to evaluate the diagnostic performance of the models. Routine magnetic resonance imaging (MRI) radiomic texture feature models generated in the same manner were used for the horizontal comparison.

**Results:**

NODDI-radiomic texture analysis based on TBV subregions exhibited the highest accuracy (although nonsignificant) in differentiating GBM from SBM, with area under the ROC curve (AUC) values of 0.918 and 0.882 in the training and test datasets, respectively, compared to necrosis (AUC_training_:0.845, AUC_test_:0.714), solid tumor (AUC_training_:0.852, AUC_test_:0.821), peritumoral edema (AUC_training_:0.817, AUC_test_:0.762), and ABV (AUC_training_:0.834, AUC_test_:0.779). The performance of the five ROI radiomic texture models in routine MRI was inferior to that of the NODDI-radiomic texture model.

**Conclusion:**

Preoperative NODDI-radiomic texture analysis based on TBV subregions shows great potential for distinguishing GBM from SBM.

**Supplementary Information:**

The online version contains supplementary material available at 10.1186/s12885-023-11718-0.

## Key points

• NODDI-radiomic texture analysis can distinguish glioblastoma multiforme from solitary brain metastasis.

• ROI division affects efficiency, and tumor bulk volume outperformed other ROIs.

• NODDI-based outperforms routine magnetic resonance imaging-based radiomic texture analysis.

**Supplementary Information:**

The online version contains supplementary material available at 10.1186/s12885-023-11718-0.

## Background

Glioblastoma multiforme (GBM) and solitary brain metastases (SBM) are the most common malignant brain tumors, and their correct identification is key for further diagnosis and treatment [1–3]. Although magnetic resonance imaging (MRI) is the main tool for differentiating between the two types of tumors, both GBM and SBM may show marked peritumoral edema and similar contrast-enhancement patterns on routine MRI, leading to great challenges in identification [4–6].

Previous studies reported that radiomics combined with routine MRI showed significant advantages in distinguishing GBM from SBM and suggested that specific imaging features are helpful in distinguishing between the two types of tumors [7, 8]. Currently, the acquisition of specific image features can be summarized into two trends: applying special MRI modalities or focusing on specific image feature types [9, 10].

Diffusion-weighted imaging (DWI) can provide a class of microscopic features related to the movement of water molecules in tissues, such as the current advanced diffusion imaging model and neurite orientation dispersion and density imaging (NODDI) [11, 12]. NODDI is a multi-spherical shell diffusion model based on the difference in the diffusion of water molecules inside and outside the cell and is more often used to characterize the difference in water diffusion between tumor infiltration and vasogenic edema [13–15].

Texture features are considered image feature types, and radiomic texture analysis is a sensitive technique that allows for a subtle assessment of the gray-scale signal intensity distribution of pixels and/or voxels, which can be used to quantify lesion irregularity and heterogeneity in tissue composition on MRI [16]. Several studies have evaluated the application of texture analysis to conventional imaging modalities for various diseases [17–21] but there are no reports on radiomic texture analysis from NODDI. We speculate that radiomic texture analysis may provide more advantages than routine MRI in distinguishing GBM from SBM.

Here, considering the sensitivity of texture features in regions of interest (ROIs), we created different ROI-based prediction models using texture features derived from NODDI. We then evaluated how well each radiomic texture analysis model could distinguish GBM from SBM and compared the prediction models for routine MRI radiomic texture analysis.

## Materials and methods

### Patients

**Fig. 1:**
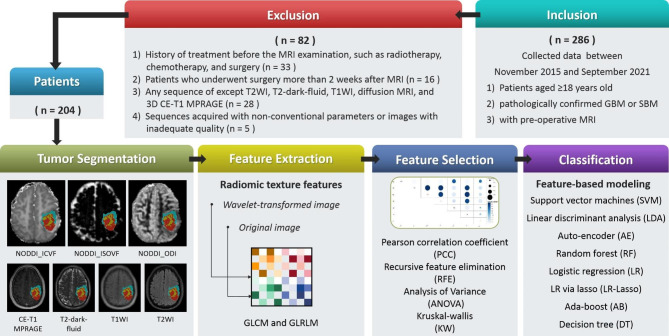
Image processing pipeline for radiomic texture analysis. GLCM, gray-level co-occurrence matrix; GLRLM, gray-level run-length matrix; GBM, glioblastoma multiforme; SBM, solitary brain metastasis

This retrospective study was approved by our institutional ethics committee, which waived the requirement for informed patient consent. The study procedures were in line with the guidelines laid out in the Declaration of Helsinki. Records from a total of 204 patients newly diagnosed with cerebral GBM or SBM between November 2015 and December 2022 were reviewed, and the inclusion and exclusion criteria listed in Fig. 1 were applied. Patients were then divided into a training dataset (diagnosed between December 23, 2015, and October 11, 2021 [n = 143]) and a time-independent test dataset (diagnosed between October 16, 2021, and December 26, 2022 [n = 61]). The demographic and clinical data are summarized in Table 1.

### MRI acquisition

All images were acquired using a 3T MR scanner (MAGNETOM Prisma; Siemens Healthcare, Erlangen, Germany) with a 64-channel head/neck coil. The structural MR protocols included axial T2-weighted imaging (T2WI), T2-dark-fluid, T1WI, three-dimensional (3D) contrast-enhanced T1 magnetization prepared by rapid gradient echo (CE-T1 MPRAGE), and DWI. The parameters of all MRI sequences are listed in Table 2.

**Table 1:** Clinical characteristics of patients whose data were included in the training and test datasets

Characteristic	Training dataset (*n* = 143)					Test dataset (*n* = 61)			*p* value
	GBM (*n* = 102)	SBM (*n* = 41)	*p* value		GBM (*n* = 44)		SBM (*n* = 17)	*p* value	
Age, years									
Mean ± SD	52.1 ± 12.0	56.5 ± 11.6	0.516		53.0 ± 9.7		55.4 ± 11.4	0.451	0.347
Sex, *n*			0.855					0.763	0.832
Male (%)	58 (56.8)	24 (58.5)			24 (54.5)		10 (58.8)		
Female (%)	44 (43.2)	17 (41.5)			20 (45.5)		7 (41.2)		
Variety of SBM, *n*									
Lung, *n*									
Adenocarcinoma (%)		28 (68.3)					13 (76.4)		
Squamous cell carcinoma (%)		2 (4.9)							
Neuroendocrine carcinoma (%)		3 (7.4)					1 (5.9)		
Small cell lung carcinoma (%)		1 (2.4)							
Poorly differentiated carcinoma (%)		1 (2.4)							
Stomach, *n*									
Adenocarcinoma (%)		1 (2.4)					0		
Kidney, *n*									
Clear cell carcinoma (%)		3 (7.4)					1 (5.9)		
Uterus, *n*									
Endometrial carcinoma (%)		1 (2.4)					1 (5.9)		
Unknown site, *n* (%)		1 (2.4)					1 (5.9)		

GBM, glioblastoma multiforme; SBM, single brain metastasis; SD, standard deviation

**Table 2:** Sequence parameters

Sequences	Slice orientation	TR/TE (ms)	Number of slices	Slice thickness	FOV (mm^2^)	Acquisition matrix	Scan time
T1WI	Axial	250.0/2.46	20	5.0 mm	220 × 220	314 × 314	37 s
T2WI	Axial	4,090.0/99.0	20	5.0 mm	220 × 220	733 × 733	34 s
T2-dark-fluid	Axial	8,000.0/81.0	20	5.0 mm	220 × 220	314 × 314	1 min 38 s
Diffusion-weighted imaging	Axial	2,500.0/71.0	60	2.2 mm	220 × 220	100 × 100	6 min 34 s
CE-T1 MPRAGE	Sagittal	2,300.0/2.32	176	0.9 mm	240 × 240	266 × 266	5 min 21 s

CE-T1 MPRAGE, contrast-enhanced T1 magnetization prepared rapid gradient echo

DWI was performed using a spin-echo echoplanar imaging sequence with the following additional parameters: six b-values (0, 500, 1000, 1500, 2000, and 2500 s/mm^2^) with diffusion encoding in 30 directions for every nonzero b-value and one for the zero b-value, and acceleration number of simultaneous multiple slices and integrated parallel acquisition technique, 3 × 2.

CE-T1 MPRAGE acquisition was performed after intravenous injection of 0.2 mL/kg of gadopentetate dimeglumine (Magnevist, Bayer Schering Pharma AG, Berlin, Germany) using a high-pressure syringe, followed by a 20-mL saline flush at the same injection rate. CE-T1 MPRAGE images were obtained after contrast agent administration and were reconstructed into 20 axial slices before use.

### Image processing

Head motion and eddy current corrections were conducted on all DW images using the Diffusion Kit Eddy tool (https://diffusionkit.readthedocs.io/). Subsequently, the NODDI metric maps were estimated directly from the DW images using NeuDilab, an in-house software developed using Python based on the free DIPY toolbox (https://www.dipy.org/). Finally, NODDI metric maps were constructed, including the intracellular volume fraction, orientation dispersion index, and isotropic volume fraction.

### MR image segmentation

**Fig. 2:**
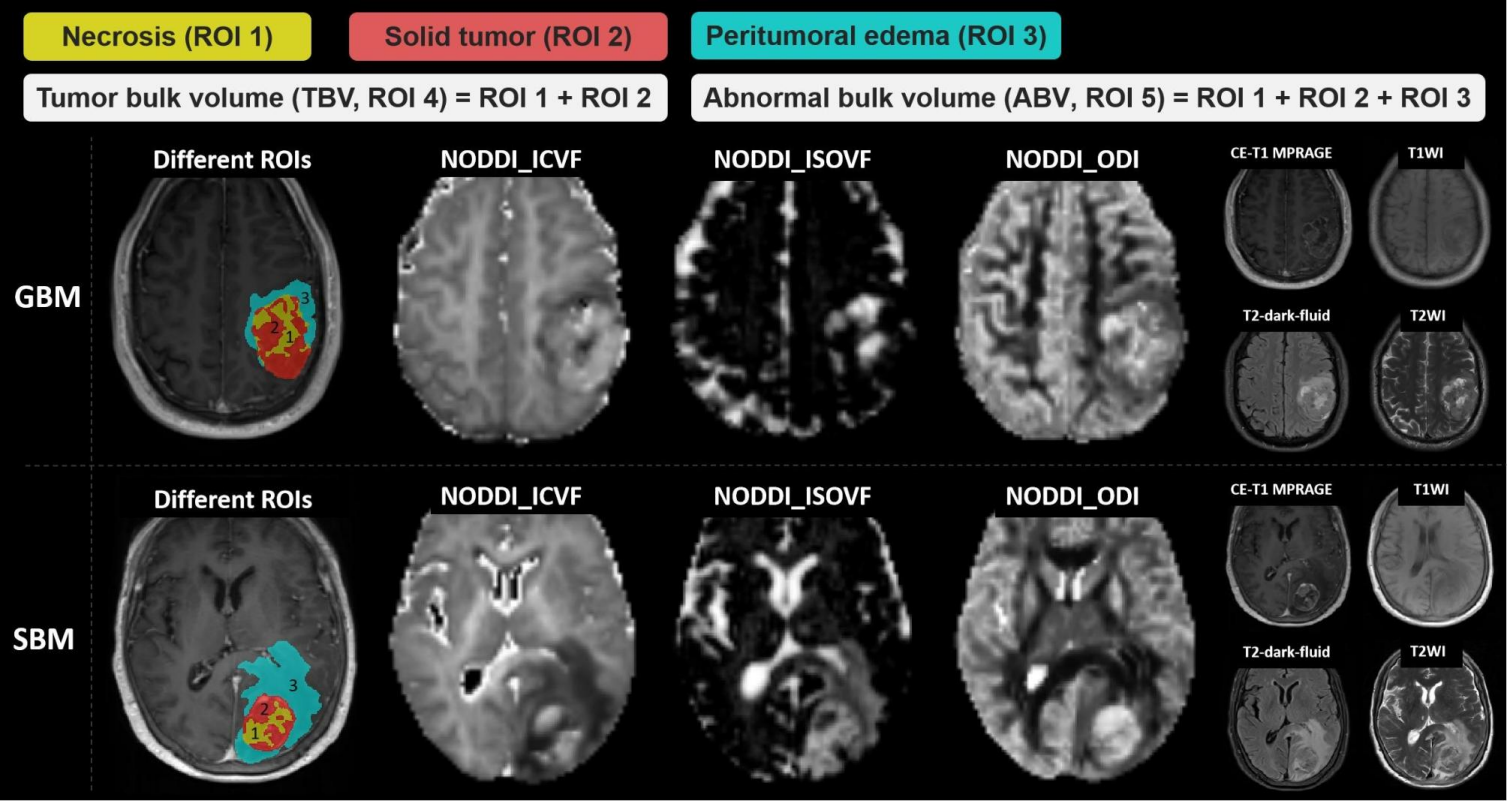
GBM and SBM derived from CE-T1 MPRAGE images for different regions of interest (ROIs) mapping and visualization. Tumor bulk volume (TBV) represents the addition of the tumor necrotic areas and solid tumor areas. Abnormal bulk volume (ABV) represents the largest area of the abnormal signal. Peritumoral edema is the difference between TBV and ABV. GBM, glioblastoma multiforme; SBM, solitary brain metastasis

MR images were first registered to T2-dark-fluid images using the open-source software ITK-SNAP (version 3.8.0; http://www.itksnap.org). Subsequently, ROIs were assessed using semi-automatic segmentation. Specifically, we constructed a deep learning model based on nnU-Net to automatically segment ROIs [22]. Details of the segmentation are presented in Supplementary Appendix E1. Five separate ROIs were defined: necrosis, solid tumor, peritumoral edema, tumor bulk volume (TBV), and abnormal bulk volume (ABV). Figure 2 shows examples of two segmentation cases based on semiautomatic segmentation.

### Radiomic texture extraction and model construction

Feature extraction, feature selection, and model building were performed using the open-source software FeAture Explorer (FAE, version 0.5.2) [23]. Based on the automatically segmented ROIs, radiomic texture features were extracted using first-order statistical functions, gray-level co-occurrence matrix (GLCM) functions, and gray-level run-length matrix (GLRLM) functions on the original NODDI parametric maps, as well as eight sub-bands of its wavelet transformation. As controls, the features of routine MR images (T2WI, T2-dark-fluid, T1WI, and CE-T1 MPRAGE) were extracted in the same manner. Radiomic texture analysis for each ROI was based on a combination of three parametric map features from the NODDI or a combination of four routine MRI features. Finally, 234 features were extracted from each parameter map of NODDI (or each type of routine MRI). Details of the extracted features are presented in Supplementary Appendix E2.

Because of the imbalanced GBM-to-SBM sample ratio (2.5:1), we applied upsampling to the training dataset. After feature extraction, all radiomic texture feature values were normalized using the min-max or Z-score method. Four feature selection methods—Pearson’s correlation coefficient (PCC), analysis of variance, recursive feature elimination, and the Kruskal-Wallis test—as well as eight classifiers—support vector machine, linear discriminant analysis, auto-encoder, random forest, logistic regression, logistic regression via Lasso, ada-boost, and decision tree—were utilized to construct texture feature prediction models for each ROI. When the PCC value of a feature pair was greater than 0.90, only one of the features was randomly retained. Five-fold cross-validation was used to determine the hyperparameters of each model. After determining the hyperparameters, all training data were retrained for the final models. The maximum number of features included in the radiomic texture analysis model construction was four. Details of the sample size and feature number estimates are displayed in Supplementary Appendix E3. The final models were determined based on the highest area under the receiver operating characteristic (ROC) curve (AUC) value in the cross-validation, and a time-independent test dataset was used to evaluate the performance of the final model. The performance of the test dataset was determined through ROC curve analysis and evaluations of accuracy, AUC, sensitivity, specificity, positive predictive value (PPV), and negative predictive value (NPV).

### Statistical analysis

Statistical analyses were performed using SPSS (version 21.0) and MedCalc (version 20.015) software. Differences in clinical characteristics between GBM and SBM were assessed using chi-square tests (or the Mann–Whitney U test, depending on the results of normality and homoscedasticity tests) and independent t-tests, as appropriate. DeLong’s test was used to assess differences in AUC values between models. Statistical significance was set at a two-sided *p* value < 0.05.

## Results

### Patient clinical characteristics

The patients’ clinical characteristics are summarized in Table 1. No significant differences were found in clinical characteristics between the patients in the training and test datasets (all *p* > 0.05). A total of 146 (71.5%) patients had GBMs, and 58 (28.5%) were diagnosed with SBM by pathological examination. The GBM rates were 71.3% (102/143) and 72.1% (44/61) for the training and test datasets, respectively, with no significant difference between the two (*p* = 0.907).

### Performance of texture feature prediction models

**Fig. 3:**
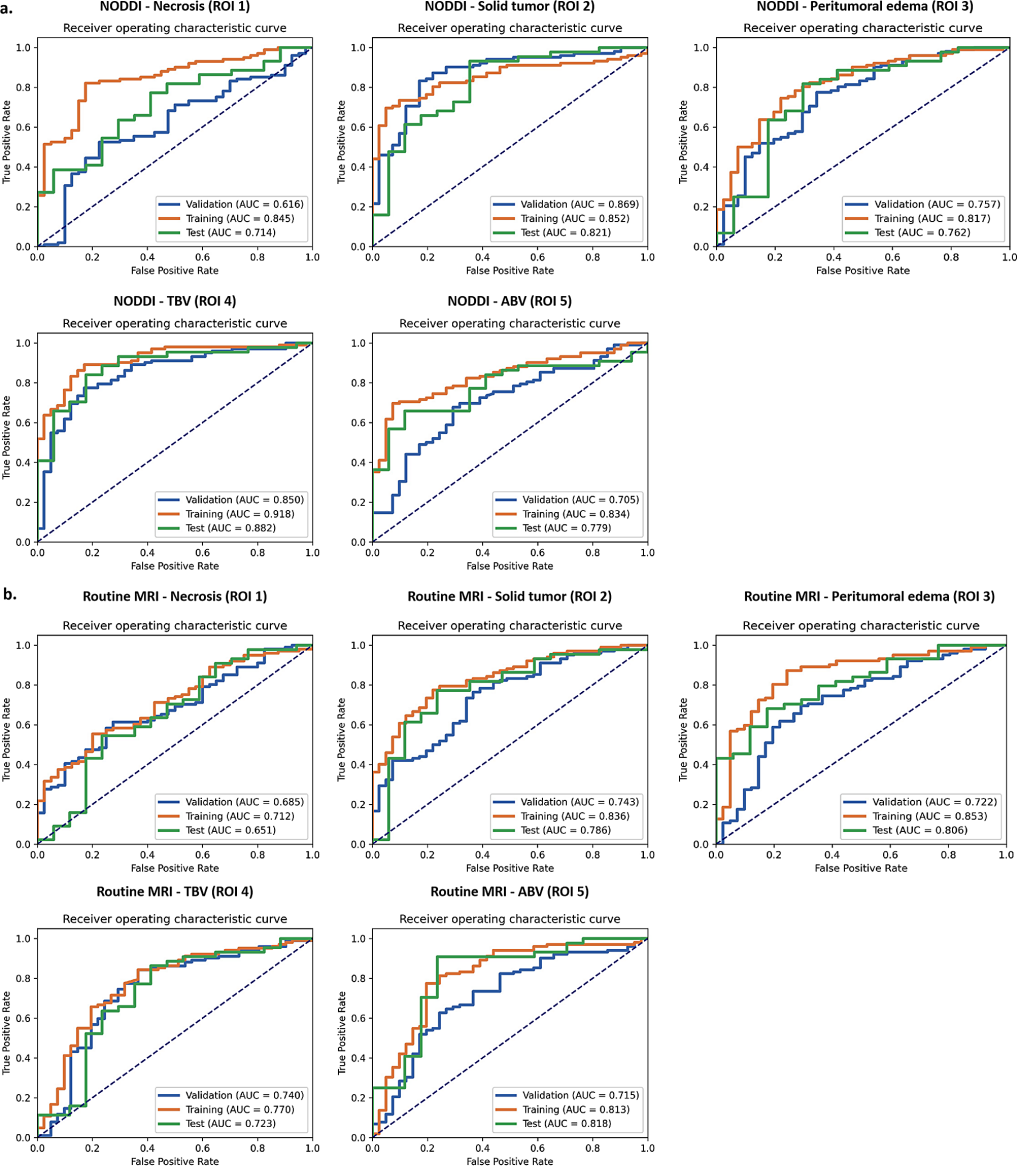
Performance of the NODDI and routine MRI radiomic texture analysis models based on five ROIs. NODDI, neurite orientation dispersion and density imaging

The NODDI-radiomic texture model based on the five ROIs performed differently when discriminating between GBM and SBM, and the TBV radiomic texture model exhibited the best performance. In the training set, we determined AUCs for the necrosis, solid tumor, peritumoral edema, TBV, and ABV texture models of 0.845, 0.852, 0.817, 0.918, and 0.834; the same values for the five models in the test set were 0.714, 0.821, 0.762, 0.882, and 0.779. Figure 3 shows the cross-validation, training set, and test set AUCs of the texture model for the five ROIs.

**Table 3:** Performance on the test dataset: NODDI and routine MRI radiomic texture model

Model	Accuracy (%)	AUC (95% CI)	Sensitivity (%)	Specificity (%)	PPV (%)	NPV (%)
NODDI-Necrosis	72.1	0.713 (0.574–0.853)	77.2	58.8	82.9	50.0
NODDI-Solid tumor	85.2	0.821 (0.695–0.946)	93.1	64.7	87.2	78.5
NODDI-Peritumoral edema	78.6	0.762 (0.610–0.914)	81.8	70.6	87.8	60.0
NODDI-TBV	83.6	0.882 (0.789–0.975)	84.1	82.3	92.5	66.7
NODDI-ABV	72.1	0.779 (0.667–0.897)	65.9	88.2	93.5	50.0
Routine MRI-Necrosis	60.7	0.651 (0.483–0.819)	54.5	76.4	85.7	39.4
Routine MRI-Solid tumor	77.1	0.786 (0.649–0.923)	77.3	76.5	89.5	56.5
Routine MRI-Peritumoral edema	72.1	0.806 (0.692–0.919)	68.2	82.4	90.9	50.0
Routine MRI-TBV	78.7	0.723 (0.561–0.885)	86.3	58.8	84.4	62.5
Routine MRI-ABV	83.8	0.818 (0.684–0.952)	90.9	76.4	90.9	76.5

95% CI, 95% confidence interval; ABV, abnormal bulk volume; AUC, area under the ROC curve; NODDI, neurite orientation dispersion and density imaging; TBV, tumor bulk volume; MRI, magnetic resonance imaging; PPV, positive predictive value; NPV, negative predictive value

The performance of the five ROI texture models on routine MRI was inferior to that of the NODDI-radiomic texture model. The AUCs of the necrosis, solid tumor, peritumoral edema, TBV, and ABV texture models were 0.712, 0.836, 0.853, 0.770, and 0.813 in the training set and 0.651, 0.786, 0.806, 0.723, and 0.818 in the test set.

**Fig. 4:**
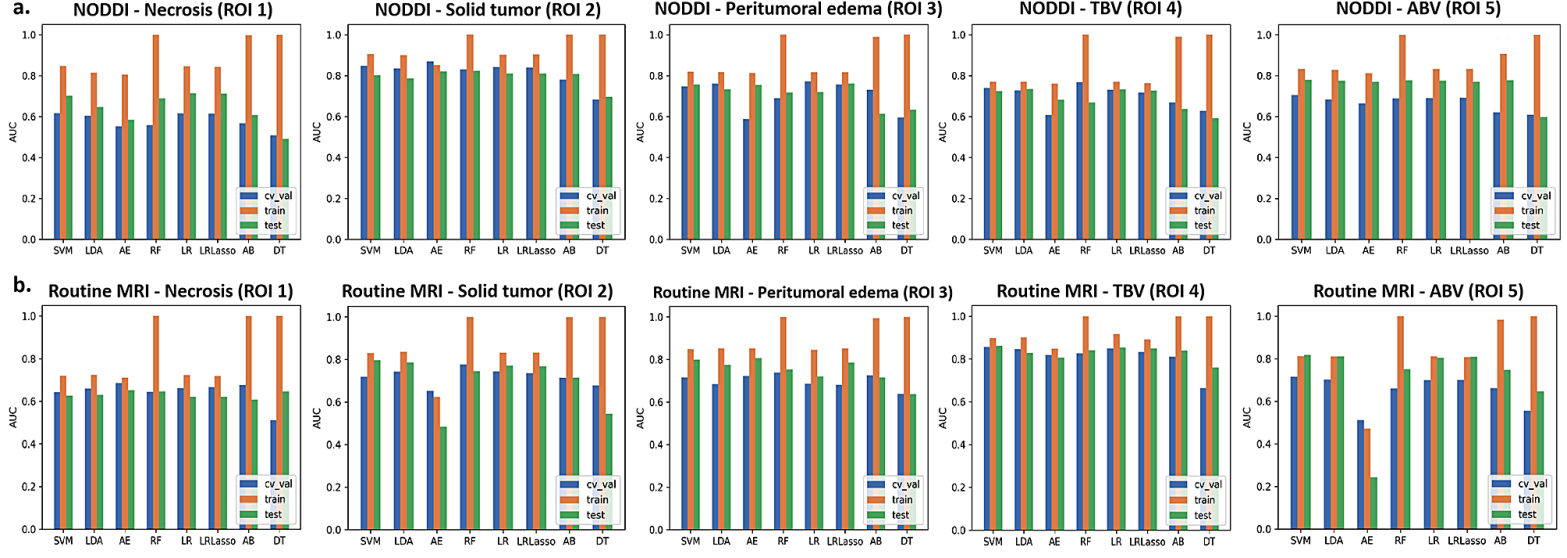
Cross-validation set, training set, and test set AUCs of the different classifiers. AUC, area under the ROC curve; ABV, abnormal bulk volume; TBV, tumor bulk volume

**Fig. 5:**
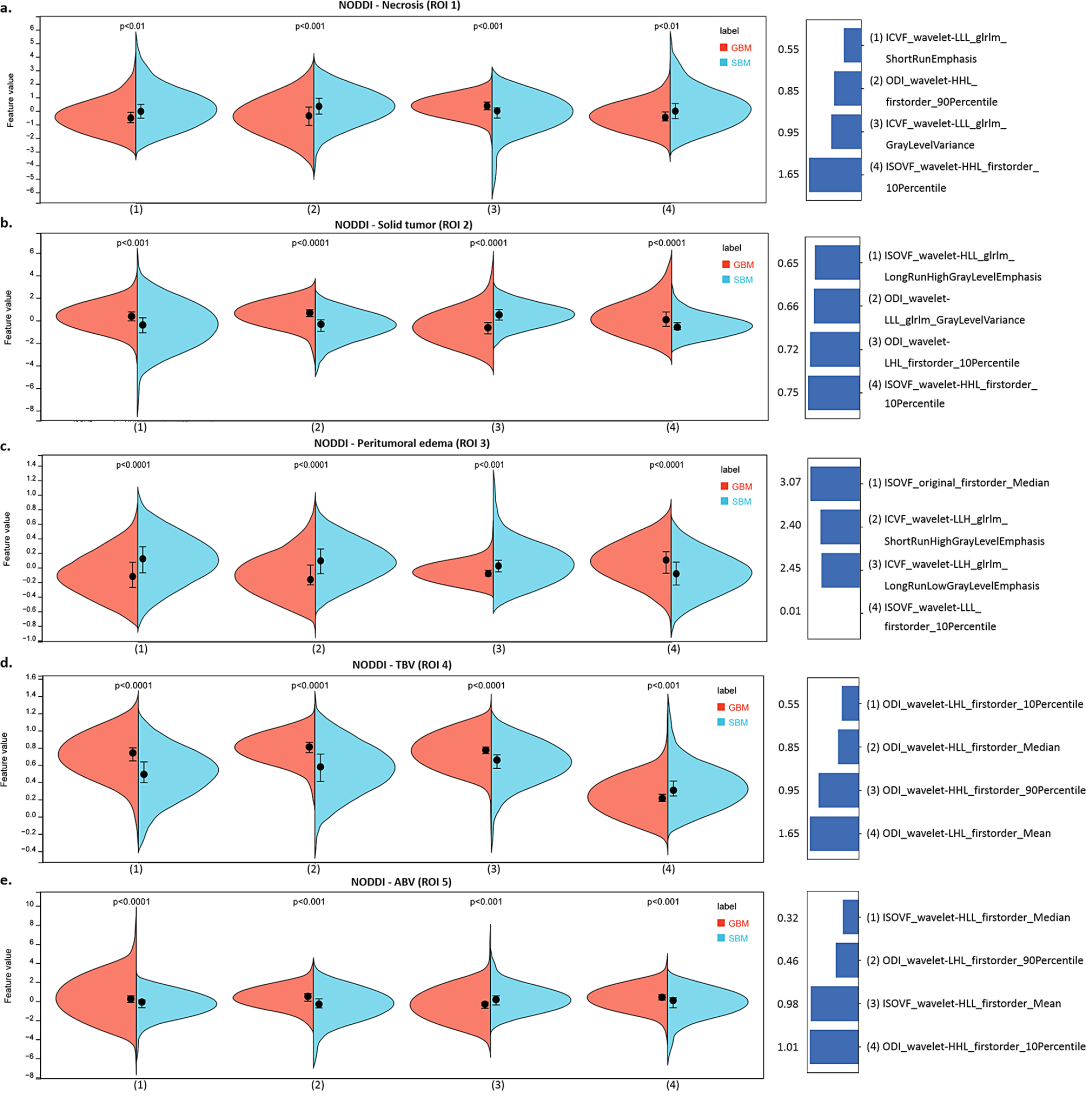
Feature distribution and contribution of the NODDI radiomic texture models

**Fig. 6:**
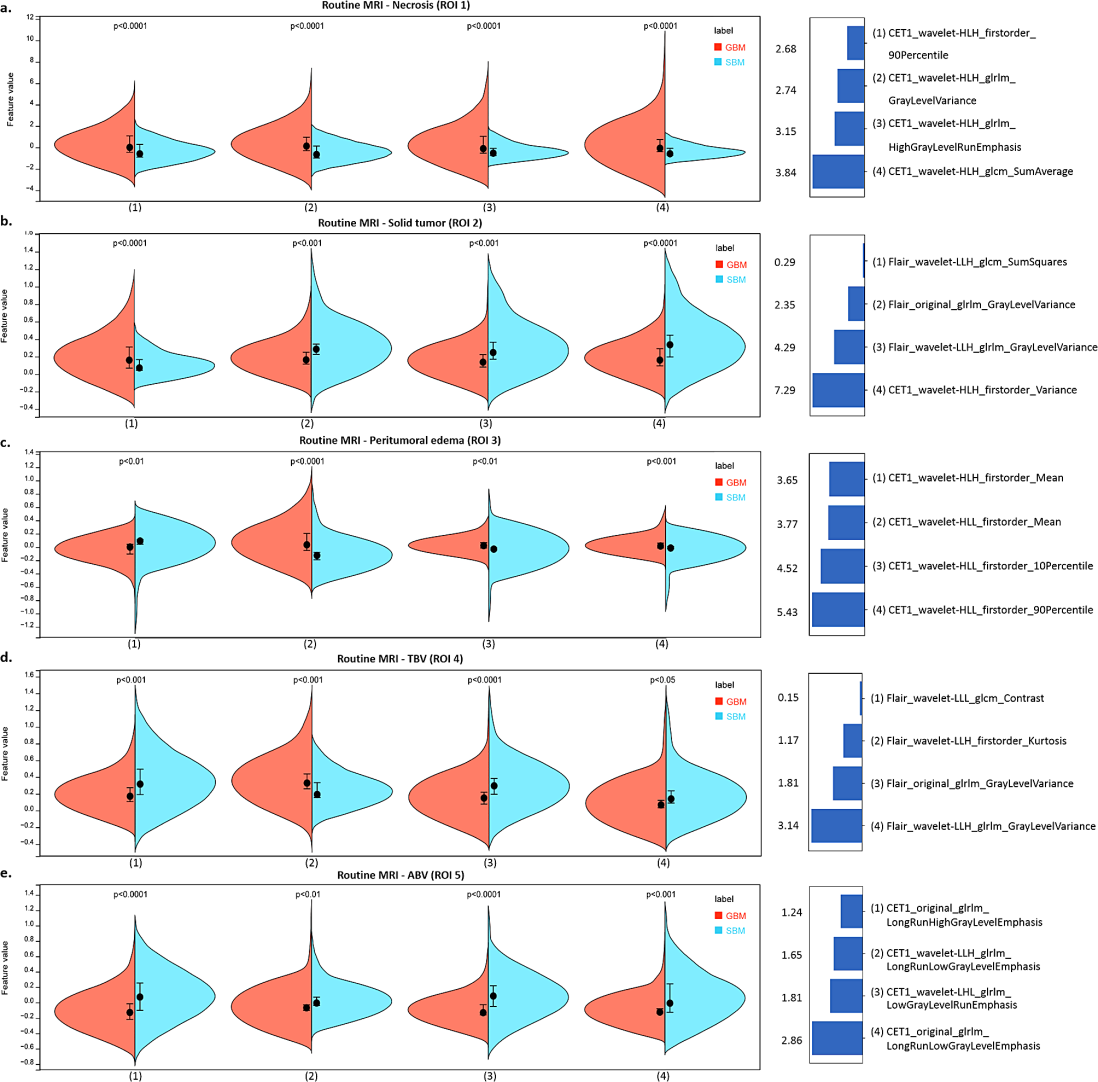
Feature distribution and contribution of the routine MRI radiomic texture models

A more detailed comparison of the evaluation indicators between the NODDI and conventional MRI radiomic texture models for the five ROIs in the test set (accuracy, sensitivity, specificity, PPV, and NPV) is provided in Table 3. The NODDI TBV texture model achieved better sensitivity (84.1%) and specificity (82.3%), proving its excellent performance on imbalanced datasets. More details on the model construction are provided in Fig. 4, showing the cross-validation set, training set, and test set AUCs for different classifiers in the determination of optimal model performance. Figures 5 and 6, and Supplementary Table 1 show the feature values, distributions and statistical correlations, contribution of features, and methods used in the key modeling steps for each model. The DeLong test results for each model are presented in Supplementary Table 2.

## Discussion

To the best of our knowledge, this is the first study that comprehensively explores the value of radiomic texture analysis based on preoperative NODDI in differentiating GBM from SBM. Our findings reveal the excellent potential and application value of NODDI-radiomic texture analysis based on the TBV for differentiating between the two types of tumors.

Analogous to histogram analysis, radiomic texture analysis is a nimble and interpretable method. Prior research has showcased the efficacy of MRI texture analysis in differentiating GBM from SBM [24, 25]. Ortiz-Ramón et al. proposed a method that combines two-dimensional texture analysis and machine learning technology to differentiate between GBM and SBM. They obtained an AUC of 0.896 by constructing a model using 32 texture features [24]. Our research, based on texture analysis, attained comparable discriminatory capability utilizing four texture features. Moreover, preceding studies proposed that routine MRI possesses restricted capacity to differentiate between the two tumor types. Han et al. developed a radiomics model on T2WI images to distinguish GBM and SBM, achieving an AUC of 0.696 [25]. Our results corroborate this assertion; although four routine sequences were combined to construct the model, only moderate discriminatory capability was ultimately achieved. Our results indicate that the NODDI texture model outperforms routine MRI texture models. The clinical benefits of NODDI over MRI include the elimination of risks associated with the use of contrast agents and a reduction in the number of parameters needed. We speculate that texture analysis based on NODDI can quantify the extent of axonal dispersion or damage within the lesion, which is very effective in distinguishing between GBM and SBM.

Various ROI settings lead to the difference in the identification ability of the model. Although specific texture features can be found in tumors and peritumoral edema, the discrimination ability of these features shows two trends. The model containing tumor regions showed a stronger ability to distinguish GBM from SBM. In fact, the tumor parenchymal area in GBM exhibits dense cell growth, heterogeneity, and nuclear atypia morphology. The tumor parenchyma of SBM exhibits relative homogeneity. This difference can be attributed to the different growth patterns of the two types of tumors. The radiomics texture analysis model based on NODDI seems to be able to distinguish the growth patterns of GBM and SBM. The pathological differences manifested by the peritumoral edema area of GBM (comprising tumor cells and new blood vessels) lead to the formation of invasive edema. In SBM, normal brain tissue is compressed by tumors, and simple vasogenic edema forms around the tumor. However, the identification efficiency of the edema area model is significantly reduced, and the radiomics texture analysis model based on NODDI cannot well distinguish invasive edema from angiogenic edema. In general, among the models based on five different ROI subregions, our NODDI-radiomic texture model based on TBV performed the best. TBV is a combination of necrosis and tumor parenchyma, and we speculate that relying on TBV guarantees the continuity and integrity of tumor texture features, leading to stronger identification ability.

Previous studies have reported progress in the ability of DWI and diffusion tensor imaging (DTI) to differentiate between GBM and SBM [26–28]. The meta-analysis conducted by Suh et al. suggests that the diagnostic abilities of DWI and DTI are limited (i.e., a pooled sensitivity of 79.8% [95% CI, 70.9–86.4%] and a pooled specificity of 80.9% [95% CI, 75.1–85.5%]), despite their wide individual sensitivity and specificity [26]. DWI and DTI are better suited as part of a multiparametric MRI protocol than as single sequences [25]. NODDI, an extension of DWI, is the only imaging modality with the potential to accurately differentiate GBM from SBM [29].

First-order statistical features are considered low-order features, whereas the GLCM and GLRLM are considered high-order features and are usually extracted from the original image or the wavelet image derived from the original image [10]. These features describe the statistical relationships between image pixels (voxels) from different perspectives and are often highly correlated and redundant [30]. Feature selection is therefore necessary. Feature selection simplifies the features used for texture analysis, excludes non-contributing and highly correlated features, reduces the redundancy and multicollinearity of candidate texture features, and facilitates the use of machine learning models for evaluation. In this study, four candidate features for building radiomic texture analysis models were identified, and the values of these reproducible features were independent of the traditional clinicopathological features.

The fifth edition of the World Health Organization classification of central nervous system tumors (WHO CNS5) emphasizes the value of molecular pathology in the diagnosis of GBM [31]. Most pathology centers need time to adapt to changes in the new WHO CNS5, thereby delaying the update of classification standards. Notably, all patients whose data were included in the present study were classified based on the 2016 WHO guidelines.

This study has, however, several limitations. First, the relatively small sample size was adequate for our tentative exploration but limits statistical power as well as the generalizability of our results. Second, samples need to be divided into training and testing datasets for internal validation. Unfortunately, we were not able to externally validate our results. Finally, it is imperative to enhance the transparency surrounding the associations between biological interpretability and advanced imaging parameters. This will enable the provision of substantial evidence for the treatment and care of tumors, as well as microscopic studies such as single-cell analysis [32, 33].

## Conclusions

Preoperative NODDI-radiomic texture analysis based on TBV shows great potential for distinguishing GBM from SBM. Further studies are required to explore the generalizability of our findings through external validation and to apply these results to clinical practice.

### Electronic supplementary material

Below is the link to the electronic supplementary material.

**Supplementary Material 1: Supplemental Appendix**. E1: ROI segmentation. E2: List of extracted radiomic texture features. E3: Sample size and featyre number estimation. **Supplemental Table 1**. Radiomic texture analysis models and features on different ROIs, and key steps in modeling. **Supplemental Table 2**. The DeLong test result in test datasets

## Data Availability

The datasets generated and/or analyzed during the current study are not publicly available due to the ongoing further studies but are available from the corresponding author (ghzhao@ha.edu.cn) on reasonable request.

## Abbreviations

ABV: Abnormal bulk volume
AUC: Area under the ROC curve
DTI: Diffusion tensor imaging
DWI: Diffusion-weighted imaging
GBM: Glioblastoma multiforme
GLCM: Gray-level co-occurrence matrix
GLRLM: Gray-level run-length matrix
MRI: Magnetic resonance imaging
NODDI: Neurite orientation dispersion and density imaging
NPV: Negative predictive value
PCC: Pearson’s correlation coefficient
PPV: Positive predictive value
ROC: Receiver operating characteristic
ROI: Region of interest
SBM: Solitary brain metastasis
T2WI: T2-weighted imaging
TBV: Tumor bulk volume
