# Supplementary material for: Radiomic texture analysis based on neurite orientation dispersion and density imaging to differentiate glioblastoma from solitary brain metastasis
Source: BMC Cancer. 2023 Dec 14;23:1231. doi: 10.1186/s12885-023-11718-0 (PMC10722697; doi:10.1186/s12885-023-11718-0)
Supplement: Supplementary file 1 — Supplementary Material 1: Supplemental Appendix. E1: ROI segmentation. E2: List of extracted radiomic texture features. E3: Sample size and featyre number estimation. Supplemental Table 1. Radiomic texture analysis models and features on different ROIs, and key steps in modeling. Supplemental Table 2. The DeLong test result in test datasets [file 12885_2023_11718_MOESM1_ESM.docx]

**SUPPLEMENTARY INFORMATION:**

**Radiomic texture analysis based on neurite orientation dispersion and density imaging to differentiate glioblastoma from solitary brain metastasis**

**Supplemental Appendix**

**E1: ROI segmentation**

*ROI segmentation -* The ROIs on multi-subregions were assessed by performing semi-automatic segmentation. First, two radiologists (10 and 15 years of experience in neuroradiology, respectively) manually assessed the ROIs with overlapping areas on subregions from routine MRI sequences. The two radiologists were blinded to the clinical information and pathological results. Subsequently, we constructed a deep learning model based on nnU-Net to automatically segment the ROIs. The ROIs independently segmented by two radiologists were merged and reviewed as the ground truth for automatic segmentation. Severe discrepancies were resolved by consensus.

ROI 1 is defined as tumor necrotic areas. ROI 2 is defined as solid tumor areas (enhanced tumors). ROI 3 is defined as peritumoral edema. ROI 4 is defined as tumor bulk volume, and represents the addition of ROI 1 and ROI 2. ROI 5 is defined as abnormal bulk volume, and represents the addition of ROI 1, ROI 2 and ROI 3. ROI 5 is represented by hyperintensity extracted from T2-dark-fluid images. Edema is the difference of ROI 4 from ROI 5.

The segmentation criteria are as follows:

1) ROIs of enhanced and unenhanced tumors in the lesion and peritumoral edema area were delineated according to axial CE-T1 MPRAGE and T1WI.

2) ROIs of peritumoral edema were delineated according to axial T2WI. Tumor intensity was lower than peritumoral edema intensity on T2WI. If the border between the tumor and peritumoral edema was unclear, it was necessary to combine CE-T1 MPRAGE, T1WI, T2-dark-fluid, and T2WI multidimensional observation to determine its boundary.

**E2: List of extracted radiomic texture features**

The extracted feature types are the same for each NODDI parametric map and each routine MRI.

| Feature type | Feature name |
| --- | --- |
| Original image;  Wavelet transformed image (8 sub-bands):   1. LLH, 2. LHL, 3. LHH, 4. HLL, 5. HLH, 6. HHL, 7. HHH, 8. LLL. | (1) Firstorder_10Percentile |
|  | (2) Firstorder_90Percentile |
|  | (3) Firstorder_Energy |
|  | (4) Firstorder_Entropy |
|  | (5) Firstorder_Kurtosis |
|  | (6) Firstorder_Mean |
|  | (7) Firstorder_Median |
|  | (8) Firstorder_Skewness |
|  | (9) Firstorder_TotalEnergy |
|  | (10) Firstorder_Variance |
|  | (11) GLCM_Contrast |
|  | (12) GLCM_Correlation |
|  | (13) GLCM_SumAverage |
|  | (14) GLCM_SumSquares |
|  | (15) GLRLM_GrayLevelVariance |
|  | (16) GLRLM_HighGrayLevelRunEmphasis |
|  | (17) GLRLM_LongRunEmphasis |
|  | (18) GLRLM_LongRunHighGrayLevelEmphasis |
|  | (19) GLRLM_LongRunLowGrayLevelEmphasis |
|  | (20) GLRLM_LowGrayLevelRunEmphasis |
|  | (21) GLRLM_RunEntropy |
|  | (22) GLRLM_RunPercentage |
|  | (23) GLRLM_RunVariance |
|  | (24) GLRLM_ShortRunEmphasis |
|  | (25) GLRLM_ShortRunHighGrayLevelEmphasis |
|  | (26) GLRLM_ShortRunLowGrayLevelEmphasis |
| 1 (Original)+8 (Wavelet)=9 | 26×9=234 |

**E3: Sample size and featyre number estimation**

According to the events per predictor variable and thumb rules, 10–15 samples are required for each predictor variable to yield a stable estimate ^[1, 2]^. For the power calculation of the validation dataset, a sample of > 11 patients was required to provide 80% power and a type I error rate of 5% ^[3]^. Our dataset included 109 patients, of whom 76 and 33 were in the training and validation datasets, respectively, meeting the sample size requirement. Specifically, in the training dataset, the minimum sample size of one type of tumor was 36; thus, the maximum number of features included in the radiomics model construction was 4.

***References***

1. Wei J, Yang G, Hao X et al (2019) A multi-sequence and habitat-based MRI radiomics signature for preoperative prediction of MGMT promoter methylation in astrocytomas with prognostic implication. Eur Radiol 29(2):877–888

3. Gillies RJ, Kinahan PE, Hricak H (2016) Radiomics: images are more than pictures, they are data. Radiology 278(2):563–577

4. Bock J (2001) Power and sample size calculations. Springer, New York 11(4):309–333

**Supplemental Tables**

**Table 1.** Radiomic texture analysis models and features on different ROIs, and key steps in modeling.

| **Imaging modality** | **Habitats** | **Steps** | **Texture features** |
| --- | --- | --- | --- |
| NODDI | Necrosis (ROI 1) | Z-score  PCC  ANOVA  LR | ICVF_wavelet-LLL_GLRLM_ShortRunEmphasis  ODI_wavelet-HHL_firstorder_90Percentile  ICVF_wavelet-LLL_GLRLM_GrayLevelVariance  ISOVF_wavelet-HHL_firstorder_10Percentile |
| NODDI | Solid tumor (ROI 2) | Z-score  PCC  Relief  AE | ISOVF_wavelet-HLL_glrimLongRunHighGraylevelEmphasis  ODL_wavelet-LLL_GLRLM_GrayLevelVariance  ODL_wavelet-LHL_firstorder_10Percentile  ISOVF_wavelet-HHL_firstorder_10Percentile |
| NODDI | Peritumoral edema (ROI 3) | Mean  PCC  ANOVA  LRLasso | ISOVF_original_firstorder_Median  ICVF_wavelet-LLH_GLRLM_ShortRunHighGrayLevelEmphasis  ICVF_wavelet-LLH_GLRLM_LongRunLowGrayLevelEmphasis  ISOVF_wavelet-LLL_firstorder_10Percentile |
| NODDI | TBV (ROI 4) | MinMax  PCC  KW  LR | ODI_wavelet-LHL_firstorder_10Percentile  ODI_wavelet-HLL_firstorder_Median  ODI_wavelet-HHL_firstorder_90Percentile  ODI_wavelet-LHL_firstorder_Mean |
| NODDI | ABV (ROI 5) | Z-score  PCC  ANOVA  SVM | ISOVF_wavelet-HLL_firstorder_Median  ODI_wavelet-LHL_firstorder_90Percentile  ISOVF_wavelet-HLL_firstorder_Mean  ODI_wavelet-HHL_firstorder_10Percentile |
| Routine MRI | Necrosis (ROI 1) | Z-score  PCC  ANOVA  AE | CET1_wavelet-HLH_firstorder_90Percentile  CET1_wavelet-HLH_GLRLM_GrayLevelVariance  CET1_wavelet-HLH_GLRLM_HighGrayLevelRunEmphasis  CET1_wavelet-HLH_GLCM_SumAverage |
| Routine MRI | Solid tumor (ROI 2) | MinMax  PCC  RFE  LDA | Flair_wavelet-LLH_GLCM_SumSquares  Flair_original_GLRLM_GrayLevelvariance  Flair_wavelet-LLH_glrim_GrayLevelVariance  CET1_wavelet-HLH firstorder_Variance |
| Routine MRI | Peritumoral edema (ROI 3) | Mean  PCC  KW  AE | CET1_wavelet-HLH_firstorder_Mean  CET1_wavelet-HLL_firstorder_Mean  CET1_wavelet-HLL_firstorder_10Percentile  CET1_wavelet-HLL_firstorder_90Percentile |
| Routine MRI | TBV (ROI 4) | MinMax  PCC  KW  SVM | Flair_wavelet-LLL_GLCM_Contrast  Flair_wavelet-LLH_firstorder_Kurtosis  Flair_original_GLRLM_GrayLevelVariance  Flair_wavelet-LLH_GLRLM_GrayLevelVariance |
| Routine MRI | ABV (ROI 5) | Z-score  PCC  ANOVA  SVM | CET1_original_GLRLM_LongRunHighGrayLevelEmphasis  CET1_wavelet-LLH_GLRLM_LongRunLowGrayLevelEmphasis  CET1_wavelet-LHL_GLRLM_LowGrayLevelRunEmphasis  CET1_original_GLRLM_LongRunLowGrayLevelEmphasis |

Note: The formulas and detailed descriptions of the features are available on the pyradiomics website (https://pyradiomics.readthedocs.io/en/stable/features.html).

**Table 2.** The DeLong test result in test datasets

| **Index** | **Model 1 vs. Model 2** | ***P* value** |
| --- | --- | --- |
| 1 | NODDI-Necrosis (ROI 1) vs. NODDI-Solid tumor (ROI 2) | 0.172 |
| 2 | NODDI-Necrosis (ROI 1) vs. NODDI-Peritumoral edema (ROI 3) | 0.634 |
| 3 | NODDI-Necrosis (ROI 1) vs. NODDI-TBV (ROI 4) | **0.019** |
| 4 | NODDI-Necrosis (ROI 1) vs. NODDI-ABV (ROI 5) | 0.483 |
| 5 | NODDI-Necrosis (ROI 1) vs. Routine MRI-Necrosis (ROI 1) | 0.595 |
| 6 | NODDI-Necrosis (ROI 1) vs. Routine MRI-Solid tumor (ROI 2) | 0.486 |
| 7 | NODDI-Necrosis (ROI 1) vs. Routine MRI-Peritumoral edema (ROI 3) | 0.337 |
| 8 | NODDI-Necrosis (ROI 1) vs. Routine MRI-TBV (ROI 4) | 0.916 |
| 9 | NODDI-Necrosis (ROI 1) vs. Routine MRI-ABV (ROI 5) | 0.222 |
| 10 | NODDI-Solid tumor (ROI 2) vs. NODDI-Peritumoral edema (ROI 3) | 0.560 |
| 11 | NODDI-Solid tumor (ROI 2) vs. NODDI-TBV (ROI 4) | 0.393 |
| 12 | NODDI-Solid tumor (ROI 2) vs. NODDI-ABV (ROI 5) | 0.545 |
| 13 | NODDI-Solid tumor (ROI 2) vs. Routine MRI-Necrosis (ROI 1) | 0.091 |
| 14 | NODDI-Solid tumor (ROI 2) vs. Routine MRI-Solid tumor (ROI 2) | 0.662 |
| 15 | NODDI-Solid tumor (ROI 2) vs. Routine MRI-Peritumoral edema (ROI 3) | 0.870 |
| 16 | NODDI-Solid tumor (ROI 2) vs. Routine MRI-TBV (ROI 4) | 0.248 |
| 17 | NODDI-Solid tumor (ROI 2) vs. Routine MRI-ABV (ROI 5) | 0.974 |
| 18 | NODDI-Peritumoral edema (ROI 3) vs. NODDI-TBV (ROI 4) | 0.220 |
| 19 | NODDI-Peritumoral edema (ROI 3) vs. NODDI-ABV (ROI 5) | 0.872 |
| 20 | NODDI-Peritumoral edema (ROI 3) vs. Routine MRI-Necrosis (ROI 1) | 0.390 |
| 21 | NODDI-Peritumoral edema (ROI 3) vs. Routine MRI-Solid tumor (ROI 2) | 0.821 |
| 22 | NODDI-Peritumoral edema (ROI 3) vs. Routine MRI-Peritumoral edema (ROI 3) | 0.575 |
| 23 | NODDI-Peritumoral edema (ROI 3) vs. Routine MRI-TBV (ROI 4) | 0.661 |
| 24 | NODDI-Peritumoral edema (ROI 3) vs. Routine MRI-ABV (ROI 5) | 0.367 |
| 25 | NODDI-TBV (ROI 4) vs. NODDI-ABV (ROI 5) | 0.163 |
| 26 | NODDI-TBV (ROI 4) vs. Routine MRI-Necrosis (ROI 1) | **0.004** |
| 27 | NODDI-TBV (ROI 4) vs. Routine MRI-Solid tumor (ROI 2) | 0.239 |
| 28 | NODDI-TBV (ROI 4) vs. Routine MRI-Peritumoral edema (ROI 3) | 0.314 |
| 29 | NODDI-TBV (ROI 4) vs. Routine MRI-TBV (ROI 4) | 0.097 |
| 30 | NODDI-TBV (ROI 4) vs. Routine MRI-ABV (ROI 5) | 0.475 |
| 31 | NODDI-ABV (ROI 5) vs. Routine MRI-Necrosis (ROI 1) | 0.218 |
| 32 | NODDI-ABV (ROI 5) vs. Routine MRI-Solid tumor (ROI 2) | 0.937 |
| 33 | NODDI-ABV (ROI 5) vs. Routine MRI-Peritumoral edema (ROI 3) | 0.737 |
| 34 | NODDI-ABV (ROI 5) vs. Routine MRI-TBV (ROI 4) | 0.557 |
| 35 | NODDI-ABV (ROI 5) vs. Routine MRI-ABV (ROI 5) | 0.662 |
| 36 | Routine MRI-Necrosis (ROI 1) vs. Routine MRI-Solid tumor (ROI 2) | 0.072 |
| 37 | Routine MRI-Necrosis (ROI 1) vs. Routine MRI-Peritumoral edema (ROI 3) | 0.137 |
| 38 | Routine MRI-Necrosis (ROI 1) vs. Routine MRI-TBV (ROI 4) | 0.594 |
| 39 | Routine MRI-Necrosis (ROI 1) vs. Routine MRI-ABV (ROI 5) | 0.176 |
| 40 | Routine MRI-Solid tumor (ROI 2) vs. Routine MRI-Peritumoral edema (ROI 3) | 0.828 |
| 41 | Routine MRI-Solid tumor (ROI 2) vs. Routine MRI-TBV (ROI 4) | 0.489 |
| 42 | Routine MRI-Solid tumor (ROI 2) vs. Routine MRI-ABV (ROI 5) | 0.749 |
| 43 | Routine MRI-Peritumoral edema (ROI 3) vs. Routine MRI-TBV (ROI 4) | 0.373 |
| 44 | Routine MRI-Peritumoral edema (ROI 3) vs. Routine MRI-ABV (ROI 5) | 0.861 |
| 45 | Routine MRI-TBV (ROI 4) vs. Routine MRI-ABV (ROI 5) | 0.221 |
